# The Val158Met polymorphism of the catechol-O-methyltransference gene is not associated with long-term treatment outcomes in carpal tunnel syndrome: A randomized clinical trial

**DOI:** 10.1371/journal.pone.0205516

**Published:** 2018-10-15

**Authors:** César Fernández-de-las-Peñas, Silvia Ambite-Quesada, Hommid Fahandezh-Saddi Díaz, Paula Paras-Bravo, Domingo Palacios-Ceña, Maria L. Cuadrado

**Affiliations:** 1 Department of Physical Therapy, Occupational Therapy, Physical Medicine and Rehabilitation of Universidad Rey Juan Carlos, Alcorcón, Spain; 2 Grupo Excelencia Investigadora URJC-Banco Santander referencia Nº30VCPIGI03: Investigación traslacional en el proceso de salud—enfermedad (ITPSE), Universidad Rey Juan Carlos, Alcorcón, Spain; 3 Department of Traumatology and Orthopaedic Surgery, Hospital Universitario Fundación Alcorcón, Madrid, Spain; 4 Department of Nursing, Faculty of Nursing, University of Cantabria, Santander, Spain; 5 Department of Neurology, Hospital Clínico San Carlos, Universidad Complutense de Madrid, Madrid, Spain; Medical University of Graz, AUSTRIA

## Abstract

**Design:**

Randomized clinical trial.

**Objective:**

To investigate the association of the Val158Met polymorphism with pain and function outcomes in women with carpal tunnel syndrome (CTS) who received surgery or manual therapy including desensitization manoeuvres of the central nervous system.

**Methods:**

A pre-planned secondary analysis of a randomized controlled trial investigating the efficacy of manual therapy including desensitization manoeuvres of the central nervous system vs. surgery in 120 women with CTS was conducted. Women were randomized to receive 3 sessions of manual therapy (n = 60) or decompression of the carpal tunnel (n = 60). The primary outcome was intensity of pain (mean pain and the worst pain), and secondary outcomes included function and symptoms severity subscales of the Boston Carpal Tunnel Questionnaire. Outcomes were assessed at baseline, and 1, 3, 6, and 12 months after the intervention. Rs4680 genotypes were determined after amplifying the Val158Met polymorphism by polymerase chain reactions. We classified subjects according to their Val158Met polymorphism: Val/Val, Val/Met, or Met/Met. The primary aim (treatment group*Val158Met*time) was examined with repeated measures ANCOVA with intention-to-treat analysis.

**Results:**

At 12 months, 111 (92%) women completed the follow-up. No interaction was observed between the Val158Met genotype and any outcome: mean pain intensity (F = 0.60; P = 0.69), worst pain intensity (F = 0.49; P = 0.61), function (F = 0.12; P = 0.88) or symptom severity (F = 0.01; P = 0.98).

**Conclusion:**

The current clinical trial did not show an association between the Val158Met polymorphism and changes in pain and function outcomes after either surgery or physical therapy in women with CTS.

## Introduction

Carpal tunnel syndrome (CTS) is a common entrapment neuropathy of the upper extremity which can cause considerable pain and disability resulting in significantly costs for the society [1]. It has been reported that the prevalence of CTS in the general population ranges from 6% to 12% [2].

Management of CTS can be conservative or surgical in nature. There is some controversy on the optimal treatment strategies for CTS since both approaches are effective, although surgery has shown to be slightly superior than localized conservative treatment at long term [3,4]. It is important to note that individuals undergoing surgery exhibit a higher rate of complications than those receiving conservative therapy (pooled RR 2.03, 1.28–3.22) [4], which may explain why several patients with CTS attempt to avoid surgery [5]. It should be also noted that most studies comparing conservative to surgical interventions have mainly applied localized procedures, e.g., ultrasound, splints, laser, or TENS, mainly focusing to the hand, as the conservative approach. Although CTS has been traditionally considered a local peripheral neuropathy, there is evidence suggesting that CTS represents a complex pain syndrome presenting an altered nociceptive pain processing [6,7], and, therefore, therapeutic approaches should include nociceptive pain rational for proper management of this condition [8].

Supporting the complexity of pain perception is the potential influence of genetic factors. The catechol-O-methyltransferase (COMT) gene is a potential genetic determinant associated to nociceptive pain processing [9]; however, its role in neuropathic pain syndromes is scarce [10]. The Val158Met single-nucleotide polymorphism (rs4680) of the COMT gene is involved in the metabolic degradation of several neurotransmitters, e.g., dopamine, norepinephrin or epinephrine [9]. The rs4680 polymorphism leads to a substitution of valine (Val) with methionine (Met) at codon 158 on chromosome 22q11 resulting in different activity levels of the gene. The presence of a Val allele results in high enzymatic activity whereas the presence of a Met allele results in low enzymatic activity [9]. In fact, people with the Val/Val genotype (higher enzymatic activity) exhibit reduced pain sensitivity than those with the Met/Met genotype (lower enzymatic activity) suggesting that this genotype could predispose for chronic pain [9]. The only study investigating a potential genetic influence in CTS found that the presence of the Met/Met genotype of the Val158Met polymorphism was associated with higher pain intensity, higher symptoms severity, and lower function in a sample of women with CTS, suggesting a regulating role of Val158Met polymorphism in the phenotypic expression of this condition [11].

A recent randomized clinical trial compared a physical therapy intervention consisting of manual therapies including desensitization maneuvers of the central nervous system with surgery on pain and function in women with CTS [12]. The results of this clinical trial showed that both interventions were similarly effective for improving pain and function at 6 and 12 months [12]. This is the first trial including a multimodal physical therapy approach based on comprehensive clinical rational of nociceptive pain mechanisms and applied to individuals with CTS. Since the Val158Met polymorphism modulates the phenotypic expression of CTS [11], we considered that there may exist a genetic influence on long-term treatment outcomes. Therefore, the aim of the current study was to investigate the association of the Val158Met polymorphism with pain and function outcomes in patients with CTS after treatment. We hypothesized that women with CTS carrying the Met/Met genotype would exhibit worse long-term treatment outcomes than those carrying the Val/Val or Val/Met genotypes independently of the intervention received.

## Material and methods

This study consisted of a pre-planned secondary analysis of a randomized clinical trial (ClinicalTrials.gov: NCT01789645) comparing the effectiveness of surgery and physical therapy in CTS [12]. The study was approved by the Hospital Universitario Fundación Alcorcón (HUFA) Institutional Review Board (PI01223-HUFA12/14).

### Participants

Consecutive women with clinical and electrophysiological findings of CTS were recruited from a regional Hospital (Madrid, Spain). Patients had to exhibit pain and paresthesia within the median nerve distribution, increasing symptoms during night, positive Tinel and Phalen signs, and electro-diagnostic findings of deficits in sensory/motor median nerve conduction according to American Association of Electrodiagnosis, American Academy of Neurology and American Physical Medicine and Rehabilitation Academy guidelines [13]. International guidelines suggest that a median nerve sensory conduction velocity less than 40 mm/s and a median nerve distal motor latency greater than 4.20ms are considered as abnormal [13]. Individuals with minimal (abnormal segmental-comparative tests), moderate (abnormal median nerve sensory velocity conduction and distal motor latency), or severe (absence of median nerve sensory response and abnormal distal motor latency) CTS were included in this study [14]. Participants were excluded if they exhibited: 1, motor or sensory deficits in the ulnar or radial nerves; 2, age >65 years; 3, surgery or steroid injections in the hand; 4, other pain syndromes affecting the upper extremity (e.g., cervical radiculopathy); 5, trauma to the neck, shoulder, or upper extremity; 6, systemic disease causing CTS (e.g. diabetes mellitus, thyroid disease); 7, other comorbid medical conditions, e.g., rheumatoid arthritis or fibromyalgia; 8, pregnancy; 9, presence of depressive symptoms (Beck Depression Inventory, BDI-II>8 points); or, 10, male gender. All participants signed an informed consent prior to the inclusion in the trial. A local human research committee approved the study project (PI01223-HUFA12/14).

### Randomization and interventions

Participants were randomly assigned to receive either physical therapy or surgery. Details on the randomization procedure have been previously published [12]. As described previously in detail [12], those patients allocated to the physical therapy group received 3 sessions consisting of different manual therapies including desensitization maneuvers of the central nervous system of 30 min duration, once per week, applied by 3 different therapists with more than 6 years of clinical experience in manual therapy approaches. Briefly, desensitization maneuvers included soft tissue techniques targeting the anatomical sites of potential entrapment of the median nerve (scalene, pectoralis minor, bicipital aponeurosis, pronator teres, transverse carpal ligament, and palmar aponeurosis), lateral glides applied to the cervical spine, and tendon and nerve gliding interventions. In addition, patients were instructed in the tendon and nerve gliding interventions as homework if necessary, as previously described [12].

Patients allocated to the surgery group underwent open or endoscopic decompression and release of the carpal tunnel conducted by experienced surgeons with at least 15 years of practice in hand surgery. Since no particular surgical procedure seems to be more effective, surgery was based on surgeon’s and patient’s preference [15]. Patients allocated to this group received the same educational session for performing tendon and nerve gliding exercises as the other group.

### Outcomes

Outcomes were assessed at baseline, and 1, 3, 6, and 12 months after the end of therapy. The primary outcome was the intensity of hand pain. An 11-point Numerical Pain Rating Scale (NPRS, 0: no pain; 10: maximum pain) was used to determine the patient’s level of hand pain and the worst level of pain experienced in the preceding week [16]. The secondary outcomes were functional status and severity subscales of the Boston Carpal Tunnel Questionnaire (BCTQ) [17]. As previously described [12], for patients with bilateral symptoms, we assigned the study hand on the basis of the more self-reported symptomatic hand; if symptoms were equivalent, the mean pain of both hands was considered.

### DNA collection and COMT genotyping

At the beginning of the trial, non-stimulated whole saliva samples were collected from each participant into collection tubes (passive drooling technique) according to the standardized procedures as previously described [11]. All subjects had abstained from any kind of vigorous exercise through the previous week. Those who smoked were asked not to do so from 2 days before the collection sampling. Patients were asked not to eat or drink or chew gum for 1 hour before the sampling. Immediately after collection, samples were centrifuged at 3000 rpm for 15 min to obtain the cell sediment and they were stored at -20° C until the analysis. We decide to use saliva instead of blood sampling because saliva collection is a non-invasive, stress-free and ethic suitable assessment method.

Laboratory technicians were blinded to the subjects’ allocation group. Genomic DNA was extracted from saliva sediments using “Genomic DNA extraction and purification Kit” (Real Molecular Biology) following the manufacturer’s instructions. The single Val158Met (rs4680) nucleotide polymorphism was genotyped using a TaqMan® Drug Metabolism Genotyping Assay on a Real Time PCR ABI Prism 7000 Sequence Detection System (APPLIED BIOSYSTEM, USA) in a Genomic Unit (Centro de Apoyo Tecnológico, Universidad Rey Juan Carlos, Madrid, Spain). The 3 possible haplotypes were associated with different fluorescent dyes to determine the identification of the different genotypes: Val/Val, Val/Met, or Met/Met. The results were derived from a G→A substitution at the following sequence:

CCAGCGGATGGTGGATTTCGCTGGC [A/G] TGAAGGACAAGGTGTGCATGCCTGA

### Data analysis

Statistical analysis was performed using SPSS software, version 21.0 (Chicago, IL, USA). Sample size was based on changes in the intensity of hand pain at 1-year follow-up as described previously in detail [12]. An intention-to-treat analysis was conducted according to the groups where patients were first allocated. Key baseline demographic variables were compared between the groups using different ANOVAs for continuous data and χ^2^ tests of independence for categorical data. To determine the effect of the Val158Met polymorphism, 5x2x3 repeated measures ANCOVAs were performed with time (baseline, 1, 3, 6, 12 months) as within-subject factor, group (physical manual therapy, surgery) and polymorphism genotype (Val/Val, Val/Met, Met/Met) as between-subject factors, and adjustment for baseline outcomes. In addition, severity of CTS according to EMG analysis was also included as potential covariable. The hypothesis of interest was the 3-way interaction treatment group*Val158Met*time. Separate ANCOVAs were performed for pain, function and symptom severity. A corrected P value of P<0.017 (Bonferroni correction) for the 3-way interaction was required to be statistically significant.

## Results

**Fig 1:**
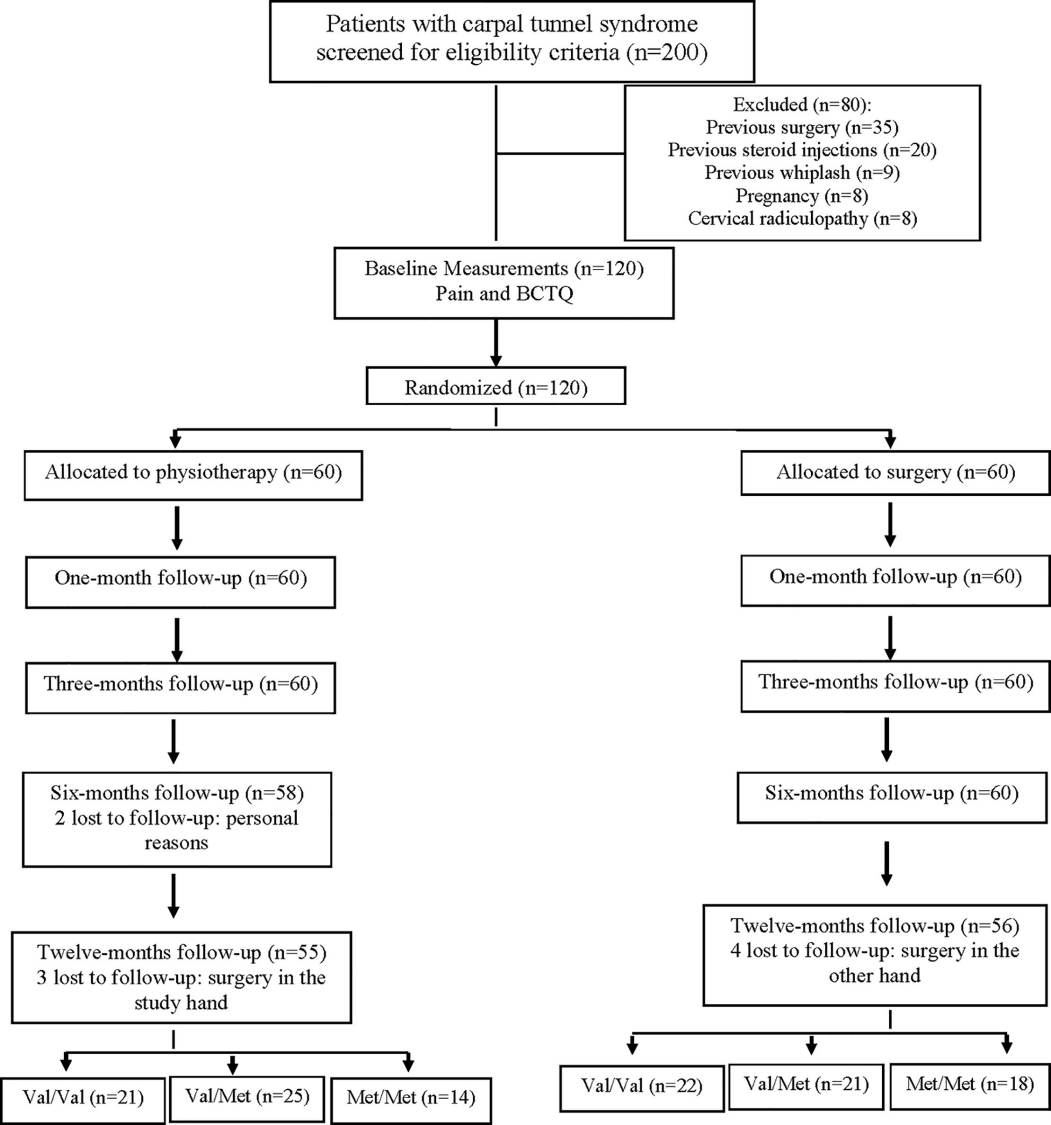
Flow diagram of patients throughout the course of the study.

Two hundred consecutive patients with CTS were screened for eligibility criteria. One hundred and twenty women (60%; mean age 47 ± 9 years old), satisfied all criteria, agreed to participate, and were randomly allocated into physical therapy (n = 60) or surgical (n = 60) groups. Of those in the physical therapy group, 14 had a Met/Met genotype (23%) whereas within those in the surgery group 17 had a Met/Met genotype (28%). **Fig 1** provides a flow diagram of subject recruitment and retention. At one-year follow-up, 111 (92%) patients provided follow-up data.

**Fig 2:**
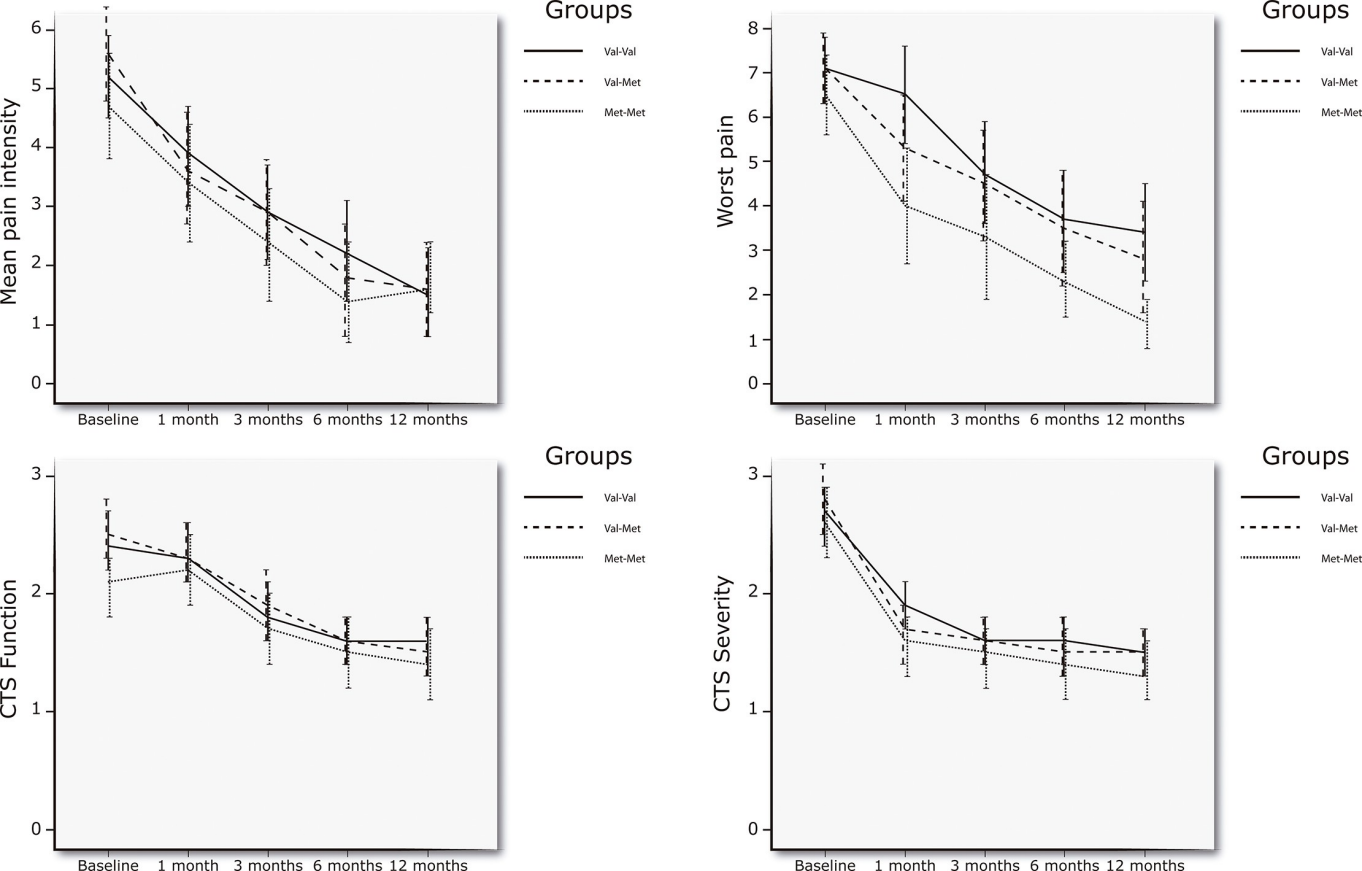
Graphical representation of the Val158Met genotype and time on pain, function and severity symptoms in the surgery group. Error bars represent 95% confidence interval of the mean scores.

**Fig 3:**
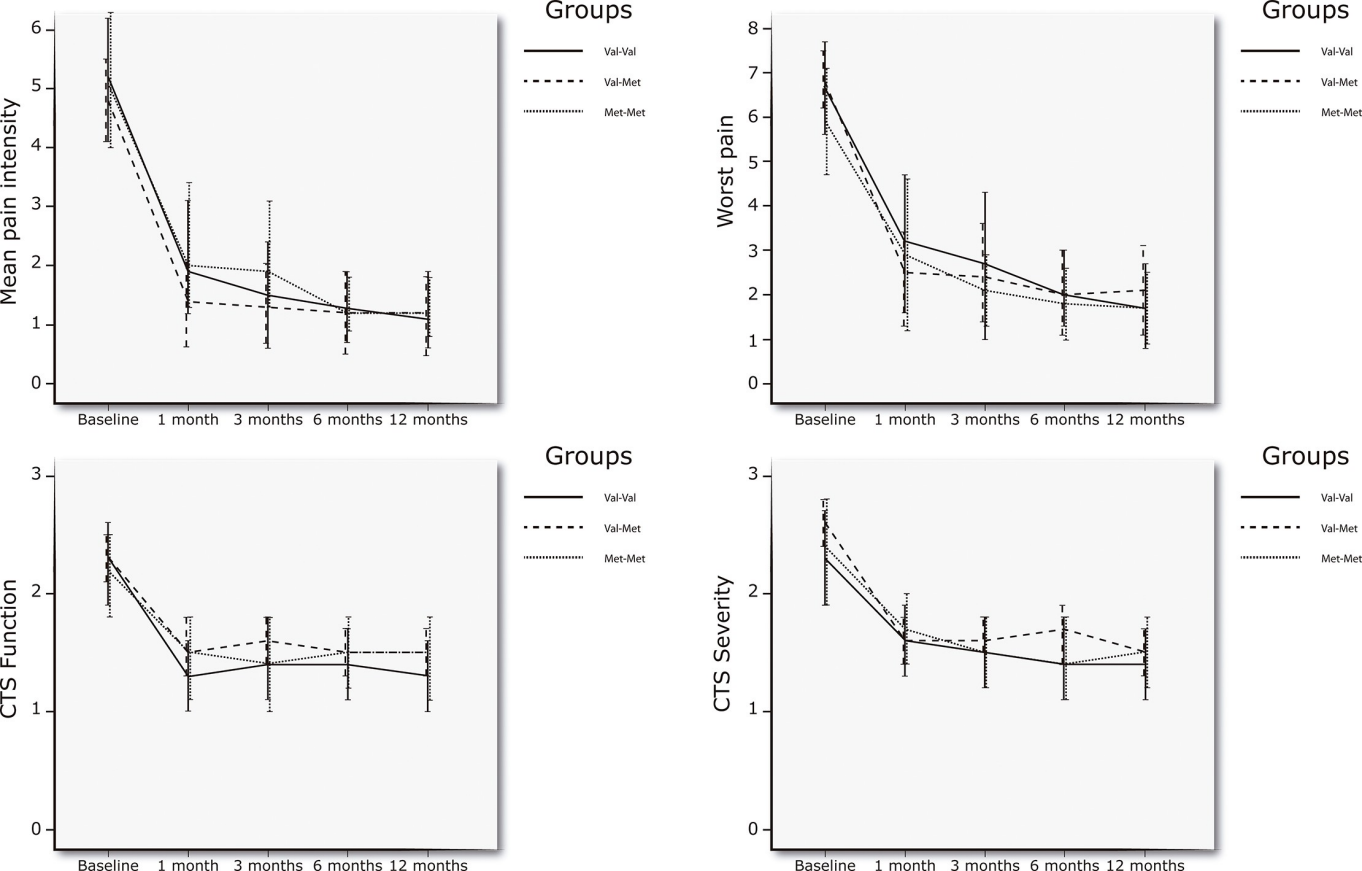
Graphical representation of the Val158Met genotype and time on pain, function and severity symptoms in the physical therapy group. Error bars represent 95% confidence interval of the mean scores.

Baseline characteristics among the groups were similar for all variables (**Table 1**). Fifty-six (93%) of the participants allocated to the surgery group received open surgery with minimal incision. The main 3-way group interaction (treatment group*Val158Met*time) for the repeated-measures ANCOVA was not statistically significant for the mean level of hand pain (F = 0.60; P = 0.69), the worst pain experienced in the last week (F = 0.49; P = 0.61), function (F = 0.12; P = 0.88) or symptom severity (F = 0.01; P = 0.98): long-term treatment outcomes were similar in all groups independently of the Val158Met genotype. The inclusion of CTS severity did not influence the results on any outcome (all, P>0.45). The graphical evolution of pain, function, and symptoms severity by Val158Met genotype can be found in **Figs 2 and 3**.

**Table 1:** Baseline characteristics by treatment assignment and Val158Met polymorphism.

	Physical Therapy Group (n = 60)			Surgery Group (n = 60)		
	Val/Val (n = 21)	Val/Met (n = 25)	Met/Met (n = 14)	Val/Val (n = 22)	Val/Met (n = 21)	Met/Met (n = 17)
**Age (years)**	45±9	47±10	46±10	47±9	48±7	46 ± 9
**Years with pain**	1.8±0.7	1.9±1.9	2.3±2.9	1.9±1.0	2.1±1.1	2.2±1.8
**Unilateral/bilateral arm distribution n (%)**						
Unilateral-right side	6 (29%)	5 (20%)	2 (14%)	6 (27%)	5 (24%)	2 (12%)
Unilateral-left side	4 (19%)	2 (8%)	2 (14%)	2 (10%)	1 (5%)	1 (6%)
Bilateral symptoms	11 (42%)	18 (72%)	10 (72%)	14 (63%)	15 (71%)	14 (82%)
**Severity n (%)**						
Minimal CTS	5 (24%)	7 (28%)	4 (28%)	5 (23%)	6 (29%)	4 (23.5%)
Moderate CTS	10 (47%)	10 (40%)	5 (36%)	10 (45%)	8 (38%)	8 (47%)
Severe CTS	6 (29%)	8 (32%)	5 (36%)	7 (32%)	7 (33%)	5 (29.5%)
**Mean intensity of the pain (NPRS, 0–10)**	5.2±1.5	4.8±1.6	5.1±1.0	5.2±2.2	5.6±1.5	4.7±2.6
**Worst pain experienced last week (NPRS, 0–10)**	6.7±1.6	6.8±1.8	5.9±1.2	7.1±2.0	7.1±1.6	6.5±2.5
**Function status scale carpal tunnel syndrome (1–5)**	2.3±0.3	2.3±0.6	2.2±0.4	2.4±0.6	2.5±0.5	2.1±0.7
**Severity status scale carpal tunnel syndrome (1–5)**	2.3±0.5	2.6±0.7	2.4±0.6	2.7±0.7	2.8±0.6	2.6±0.7

Data are expressed as number (percentage) for categorical variables and as means ± standard deviation for continuous variables

## Discussion

Our results showed that variations in the Val158Met genotype were not associated to long-term treatment outcomes in pain and function in a sample of women with CTS who received surgery or physical manual therapy. The Val158Met polymorphism has been found to modulate the response to therapy in some conditions; for instance, the effects of antipsychotic medication in schizophrenia and schizo-affective disorder patients [18], opioid consumption in postoperative nephrectomy patients [19], or the efficacy of morphine in cancer patients with pain [20]. Our study is the first one investigating the potential influence of the Val158Met polymorphism in long-term treatment outcomes in patients with CTS. We did not find a significant influence on the outcomes suggesting that COMT gene activity may be not as relevant for CTS as in other conditions. In fact, our results agree with another recent study where no genetic influence was observed in long-term treatment outcomes in individuals with chronic low back pain [21]. It is possible that the effects of physical interventions, e.g., surgery or physical therapy, have little potential genetic influence and that the Val158Met polymorphism could be more associated with pharmacological interventions. Future studies should investigate this hypothesis.

Another potential explanation for the lack of influence on the outcomes may be related to the fact that the Met allele of the Val158Met polymorphism has been associated with augmented cortical processing in individuals with chronic pain supporting the notion that COMT influence is more relevant in people with already heightened sensitization [22]. Since central sensitization is a common feature of CTS not associated with electro-diagnostic findings [23], it is plausible that Val158Met polymorphism modulation would be more associated to neuro-physiological rather than to clinical outcomes. Future trials investigating the genetic influence on changes in nociceptive pain responses in people with CTS are required.

Finally, it is interesting to note that 25% of our sample exhibited Met/Met genotype, which agrees with previous data [11]. Yet, clinical data were not significantly different among Val/Val, Val/Met or Met/Met genotypes, which is contrary to previous findings [11]. Future studies including greater sample sizes are needed to further determine the role of the Val158Met polymorphism in CTS to confirm or refute these hypotheses.

This is the first study investigating the association of the Val158Met polymorphism and long-term treatment outcomes in CTS, but the results should be considered based on potential strengths and limitations which has been described previous in detail [12]. A major strength was that we compared surgery to a well-defined non-surgical, multimodal manual therapy approach. The physical therapy program included manual therapies that could potentially benefit patients based on pain neurophysiology and current nociceptive theories of CTS. A second strength was that multiple physiotherapists and surgeons were involved in either treatment group, although most patients were derived from local regional hospitals. Nevertheless, it is important to consider that most patients received an open surgical decompression; therefore, a comparison between surgical procedures was not possible. Third, the trial had high retention rates at 12-month follow-up. However, we must also recognize some limitations. First, we only included women with CTS. We do not know if the same findings would be found in men with CTS. Second, the role of psychological variables such as depression, anxiety, or sleep disorders, and neuro-physiological outcomes, i.e., quantitative sensory testing, has not been investigated. Third, the optimal dosage for conservative treatment has not been established, and the outcomes might be different with different number or frequency of treatment sessions. Finally, we only investigated the rs4680 single nucleotide polymorphism of the Val158Met gene. Future studies should investigate the influence of a greater number of polymorphisms in the therapeutic response of CTS patients.

## Conclusions

The current clinical trial found that variations in the Val158Met polymorphism were not associated with long-term changes in pain and function in women with CTS receiving surgery or physical manual therapy.

## Supporting information

S1 Text CONSORT checklist. (DOC)

S2 Text Study protocol. (DOC)
