# Supplementary material for: The Val158Met polymorphism of the catechol-O-methyltransference gene is not associated with long-term treatment outcomes in carpal tunnel syndrome: A randomized clinical trial
Source: PLoS One. 2018 Oct 15;13(10):e0205516. doi: 10.1371/journal.pone.0205516 (PMC6188786; doi:10.1371/journal.pone.0205516)
Supplement: S2 Text — (DOC) [file pone.0205516.s002.doc]

**Provisional Title: Physical Manual Therapy versus Surgery for Carpal Tunnel Syndrome: a Randomized Parallel-group Trial**

**Full Protocol submitted to Institutional Review Board of the Hospital Universitario Fundación Alcorcón (HUFA) dated**

**10- September-2012**

This supplement contains the following items:

1. Original protocol submitted to the Institutional Review Board

2. Original statistical submitted to the Institutional Review Board

This protocol summarizes the background including the rational for conducting this study, the objective and hypothesis and the methods used for that purpose. This trial protocol gives readers additional information about the trial conducted. The authors state that no substantive changes were conducted into the protocol throughout the study.

The protocol was approved on December 2012 with the following reference number: PI01223-HUFA12/14

**PROTOCOL**

**Background**

Carpal tunnel syndrome (CTS) is the most common nerve entrapment of the upper extremity mainly featured by a compression of the median nerve at the carpal tunnel in the wrist. Papanicolaou et al reported a prevalence of 3.72% in the general American population (1). Women are most often affected reporting an annual incidence of 139 per 100,000 compared to 67 cases per 100,000 in males (women/men ratio 2:1) (2). The societal burden of CTS is substantial since these patients had several income lost due to pain (3).

Treatment approaches for individuals with CTS mainly include conservative or surgical procedures; however, their scientific evidence had exhibited conflicting results. For instance a recent Cochrane Review investigating the effectiveness of hand exercises concluded that there is limited and low quality evidence of the benefit for exercise and mobilisation interventions for CTS (4). Another Cochrane Review found that surgical treatment relieves symptoms significantly better than hand splinting; but more research is needed to determine whether this conclusion can be applied to people with mild symptoms and whether surgical treatment is better than steroid injection (5).

The most updated review comparing surgical versus conservative management showed that both approaches may be effective for CTS, but surgical treatment seems to be slightly superior, showing moderate effect sizes, than conservative interventions at long-term (6). In fact, Huisstede et al found that evidence supports the effectiveness of conservative interventions at short-term, but there is a lack of evidence on mid-term and long-term effects in CTS (7). Therefore, since most trials have included short-term effects of conservative treatments, high quality randomized clinical trials determining the efficacy of conservative interventions at mid-term and long-term results are needed. This assumption would be further supported by the fact that 61% of the individuals with CTS try to avoid surgery (8) and they typically prefer conservative management as first therapeutic option because of the higher rate of complications associated to surgery (6).

Before design a randomized clinical trial comparing the effectiveness of physical therapy versus surgery, we should analyze current advances in pain neurophysiology in this condition. CTS has been primarily considered a peripheral neuropathy localized at the carpal tunnel; therefore, previous studies comparing the use of physiotherapy and surgery applied localized treatments, including exercises, mainly focused to the hand (4-7). In addition, most of the studies included in previous reviews have used physical modalities different to manual therapy, such as hand splints (9,10), or laser (11). The only trial including manual therapies, particularly exercises, into a multimodal treatment approach was that one conducted by Jarvik et al (8). Interestingly, this study only found small differences between surgery and conservative management (8).

Recent evidence suggests that CTS is a complex syndrome showing sensitization of the central nervous system (12,13). In fact, these sensitization mechanisms are independent of electro-diagnostic findings and seem to play a potential relevant role in the development of chronic CTS (14). The presence of these sensitization processes would have an impact on conservative interventions, particularly manual therapies that should be applied to these patients. This hypothesis would be supported by preliminary data suggesting that manual physical therapies can modulate sensitization mechanisms by integrating neurophysiology of pain into their rationale (15,16). No study has applied manual therapy interventions with this consideration in patients with CTS. We believe that a high quality randomized clinical trial comparing the effectiveness of this physical manual therapy approach and surgery would help to improve the conservative management of CTS.

**Objective**

The aim of our randomized clinical trial is to determine if those manual therapies including desensitization maneuvers of the central nervous system would be effective for improving pain and function outcomes in women with CTS at short, medium and long-term follow-up periods.

**Hypothesis**

Our main hypothesis is that CTS subjects receiving manual therapies including desensitization maneuvers of the central nervous system would exhibit similar outcomes in function and pain than those patients receiving surgery at all follow-up periods.

**Study Design**

A randomized parallel-group trial will be conducted according to the CONSORT statement. The protocol has been included in the database [**https://register.clinicaltrials.gov**](https://register.clinicaltrials.gov/)with the registry number NCT01789645.

**Participants**

Women diagnosed with CTS from local regional Hospitals in Madrid (Spain) will be screened for eligibility criteria. Participants will be diagnosed according to both clinical and electrophysiological findings. To be eligible, patients should exhibit all the following signs: pain/paresthesia in the median nerve distribution, increasing symptoms during the night, positive Tinel sign, and positive Phalen sign. Symptoms should have for at least 12 months. Further, the electro-diagnostic examination had to reveal deficits of sensory and motor median nerve conduction according to international guidelines of the American Association of Electrodiagnosis, the American Academy of Neurology (AAEAAN) and American Physical Medicine and Rehabilitation Academy (APMR) (17). A median nerve sensory conduction velocity <40 mm/s and a median nerve distal motor latency >4.20 ms are considered as abnormal (17). Patients will be classified according to the classification of Padua et al (18): minimal (abnormal segmental-comparative tests only), moderate (abnormal median nerve sensory velocity conduction and distal motor latency), and severe (absence of median nerve sensory response and abnormal distal motor latency) CTS.

Participants will be excluded if they exhibit any of the following criteria: 1, any sensory/motor deficit in the ulnar or radial nerves; 2, age >65 years; 3, previous surgery or steroid injections; 4, multiple diagnoses on the upper extremity (co-existing cervical radiculopathy); 5, neck, shoulder, or upper extremity trauma; 6, systemic disease causing CTS (diabetes mellitus, thyroid disease); 7, musculoskeletal medical conditions, e.g., rheumatoid arthritis or fibromyalgia; 8, pregnancy; 9, depressive symptoms (Beck Depression Inventory, BDI-II>8 points); or, 10, male gender.

**Ethical Aspects**

All subjects will be informed of the study without knowing the real aim. They will sign the informed consent prior to their inclusion in the study. The study design will be conducted according to Helsinki Declaration.

**Allocation**

Patients will be randomly assigned to receive either physiotherapy or a surgical procedure. Concealed allocation is conducted using a computer-generated randomized table of numbers created prior to the start of the data collection by a clinical researcher not involved in the recruitment and/or treatment of patients. Individual and sequentially numbered index cards with the random assignment have been prepared. The index cards will be folded and placed in sealed opaque envelopes. A second researcher will open the envelope and proceed with treatment according to the group assignment. We will blind clinicians who obtain follow-up information to patient allocation.

**Physical Manual Therapy Intervention**

Patients allocated to the physical therapy group will receive 3 treatment sessions of manual therapies including desensitization maneuvers of the central nervous system of 30- minute duration, once/week. The techniques will include soft tissue mobilization and nerve/tendon gliding exercises. All physiotherapy treatment sessions will be applied by different therapists with 8 years of clinical experience working with this population each one.

The desensitization maneuvers include manual techniques directed at anatomical sites of potential entrapment of the median nerve (19). The session will include soft-tissue manipulation, myofascial release, stretching, and cross-fiber friction over the muscular interfaces related to the median nerve: scalenes, pectoralis minor, bicipital aponeurosis, pronator teres, transverse carpal ligament, and palmar aponeurosis. All these muscular interfaces are explored by the clinician and treated according to the following findings: pain on palpation and reproduction of sensory or motor symptoms of the patients. Other interventions targeting the neck (lateral glides applied to the cervical spine) will be also applied.

Finally, passive nerve/tendon gliding exercise targeted to the median nerve will be applied (20). This intervention intends to produce a gliding movement of the nerve and tendon in relation to their adjacent soft muscle tissues and involves the application of joint movements to the targeted structure proximally while releasing movement distally, followed by a reverse combination (21). The sequence of the nerve/tendon gliding exercise will be as follows: shoulder girdle depression, gleno-humeral abduction and lateral rotation, supination of forearm, wrist, thumb, and finger extension. In this position, concurrent elbow flexion and wrist extension is alternated dynamically with concurrent elbow extension and wrist flexion. The therapist alternates the combination of movement depending on the tissue resistance. Speed and amplitude of movement are adjusted such that no pain was produced during the technique. The intervention will be completed over 5-10 minutes in 2 sets of 5min each with 1-min rest between sets.

The third last treatment appointment will include an educational teaching session of the tendon/nerve gliding interventions for doing as homework if necessary. Patients will be asked for not modifying any work or activity levels.

**Standardization of the Physical Manual Therapy Intervention**

We will standardize the physical manual therapy intervention to be homogeneous in the management of the patients. Prior to initiation of recruitment, a professional panel of physical therapists will develop the manual therapies to be included in the protocol and a detailed standardized document. This document will be used as coadjutant help during the sessions that physical therapists will receive as training. Dr. Fernández-de-las-Peñas will conduct the training sessions with two assistants before starting the study collection to perform quality assurance.

**Surgery intervention**

Patients randomly allocated to the surgery group will undergo open or endoscopic decompression and release of the carpal tunnel according to international guidelines. For pragmatic reasons and because no clear evidence supports any particular surgical procedure, surgery will be conducted based on surgeon’s and patient’s preference (22) All surgeons are highly experienced with at least 15 years of practice and habitual practices focusing on hand surgery. Surgeons will refer patients for hand therapy after operation as their usual routine if necessary. Patients allocated to this group will also receive the same educational session for performing the tendon/nerve gliding exercises as the physiotherapy group.

**Outcome Measures**

Clinical records of all patients will include questions regarding the location of the symptoms, aggravating/relieving factors, intensity, duration of the pain and previous treatments. They also complete the BDI-II for assessing symptoms of depression. The outcomes include pain, function, symptoms severity, and self-perceived improvement.

The intensity of pain will be assessed with an 11-point Numerical Pain Rating Scale (NPRS, 0: no pain; 10: maximum pain) (23). We will assess the patients’ current level of pain and the worst level of pain experienced in the preceding week. Farrar et al determined that a change of 2 points or a 30% decrease in pain intensity from baseline can be considered as a minimal clinically important difference (MCID) for the intensity of pain in patients with chronic conditions (24). For patients with bilateral symptoms, we designated the study hand on the basis of the more self-reported symptomatic hand; if symptoms were equivalent, the mean pain of both hands was considered.

The Spanish version (25) of the Boston Carpal Tunnel Questionnaire (26) (BCTQ) will be used to assess function and severity of the disease. This questionnaire evaluates 2 domains: a) the functional status scale assessing ability to perform 8 common hand-related tasks; b) the symptom severity scale including 11 items assessing pain severity, numbness, and weakness at night and during the day. Each question is answered on a 5points scale (1: no complaint; 5: severe complaint). Higher scores indicate greater severity or worse function. This questionnaire has been shown to be valid, reliable, and responsive for individuals with CTS (27). A change of 0.74 points in the function subscale and 1.14 points in the symptom subscale can be considered as MCID of the BCTQ (28).

Finally, self-rated improvement will be assessed with a Global Rating of Change (GROC), which consists of a 15-point scale ranging from -7 (a very great deal worse) to +7 (a very great deal better) (29). Descriptors of worsening or improving are assigned with values ranging from -1 to -7 and +1 to +7, respectively (30). It has been reported that scores of +4 and +5 are indicative of moderate changes in patient status, whereas scores of +6 and +7 indicate large changes in the status of the patient.

Pain and function will be assessed at baseline, and 1, 3, 6, and 12 months after the end of therapy, whereas GROC will be assessed at 6 and 12 months by an assessor blinded to the subject’s allocation.

**Blinding**

Subjects, treating physical therapist and surgeons will not be blinded to intervention assignment. The Principal Clinical Investigator, Dr César Fernández-de-las-Peñas PhD will be blinded to crossover information.

**Sample size determinations**

We determined two sample size determination based on pain and function outcomes. For pain intensity, sample size calculation was calculated to detect treatment differences of 2.0 units on pain (MCID), assuming a standard deviation of 3.0, a 2-tailed test, an alpha level (α) of 0.05 and a desired power (β) of 90%. The estimated desired sample size with pain intensity as the main outcome was calculated to be at least 50 subjects per group. A dropout rate of 15% was expected, so around 60 patients should be included in each group.

For function, sample size calculation was determined to detect between-groups treatment differences of 0.5 points (MCID) on function subscale, assuming a standard deviation of 0.7, a 2-tailed test, an alpha level (α) of 0.05 and a desired power (β) of 90%. The estimated desired sample size with function as the main outcome measure was calculated to be at least 45 subjects per group. A dropout rate of 15%-20% was expected, so around 60 patients should be included in each group.

We decided to include pain as the main outcome in this study for the following reasons: 1, pain within the median nerve-related areas is the most common symptom experienced by patients with CTS (31); 2, pain is mainly determined by the activity of peripheral nerve nociceptors and continuous nociceptive afferent bombardment from the nerve nociceptors can lead to central and peripheral sensitization mechanisms observed in these patients (32).

**Related logistical issues**

In both group, one researcher will call individuals by telephone every month for the first 3 months of the study to provide answer questions, to assess compliance with medical appointments (within the surgical group), and to identify adverse effects of interventions. After three months, the researcher will call participants each 2 months for the remainder of the follow up period (during 9 months), particularly for remember the consecutive appointments for the study.

**Statistical Analysis**

Statistical analysis will be done using SPSS software, version 18.0 (Chicago, IL, USA) and it will be conducted according to intention-to-treat analysis for patients in the group to which they were allocated. Baseline demographic and clinical variables will be compared between groups with independent Student t-tests and χ2 test of independence. The primary evaluation will be repeated measured analyses of covariance (ANCOVA) with time as the within-subject factor and group as the between-subject factor and adjusted for baseline outcomes for evaluating between-group differences in outcomes. We will use χ2 tests to compare self-perceived improvement at 6 and 12 months in both groups. To enable comparison of effect sizes, standardized mean score differences (SMDs) will be also calculated by dividing the mean score differences between groups by the pooled standard deviation.

**Pre planned Secondary Analysis**

The existence of individuals’ differences in the response to painful stimuli suggests that potential genetic factors can be involved in nociceptive modulation. The catechol-O-methyltransferase (COMT) gene is one of the several potential genetic determinants for nociceptive processing; however, its role in chronic pain remains still controversial. The Val158Met single-nucleotide polymorphism of the COMT leads to a substitution of valine (Val) with methionine (Met) at codon 158 on chromosome 22q11. This enzyme is involved in the metabolic degradation of several neurotransmitters such as dopamine, norepinephrin, and epinephrine. Previous studies have tried to identify the association of Val158Met genotype with the presence of chronic musculoskeletal pain; however, the results are conflicting. The only study investigating genetic influence in CTS found that the presence of the Met/Met genotype of the COMT gene was not a risk factor for the development of CTS, but it was associated with worse clinical pain presentation of the patients: higher intensity of pain, higher symptoms severity, and lower function (33). These recent results would suggest a regulating role of Val158Met polymorphism in the phenotypic expression of CTS. We do not know if this genotype can be also associated with treatment response.

The Val158Met polymorphism has been found to modulate treatment efficacy of morphine in cancer patients with pain. The presence of the Met/Met genotype induces less consumption of morphine than the Val/Val genotype (34). No previous study has investigated the potential influence of the Val158Met genotype in treatment outcomes in CTS. Therefore, a secondary analysis will be conducted to investigate the association of the Val158Met polymorphism with pain and function in CTS. We hypothesized that women with CTS carrying Met/Met genotype would exhibit worse long-term treatment outcomes than those carrying the Val/Val or Val/Met genotypes.

# DNA Collection and COMT Genotyping

At the beginning of the trial, non-stimulated whole saliva samples will be collected from each participant into collection tubes (passive drooling technique) according to the standardized procedures. The following instructions will be given to participants for saliva collection: 1, all participants will be abstained from any kind of vigorous exercise through the previous week; 2, those who smoke will be asked not to do so from 2 days before the collection sampling; 3, they will be asked not to eat or drink or chew gum for 1 hour before the sampling. We will use saliva instead of blood sampling because saliva collection is a non-invasive, stress-free and ethic suitable assessment method.

Immediately after collection, samples will be centrifuged at 3000 rpm for 15 min to obtain the cell sediment and they were stored at -20º C until the analysis. Genomic DNA will extracted from saliva sediments using “Genomic DNA extraction and purification Kit” (Real Molecular Biology) following the manufacturer’s instructions. The single Val158Met (rs4680) nucleotide polymorphism will be properly genotyped using a TaqMan® Drug Metabolism Genotyping Assay on a Real Time PCR ABI Prism 7000 Sequence Detection System (APPLIED BIOSYSTEM, USA) in a Genomic Unit (Centro de Apoyo Tecnológico, Universidad Rey Juan Carlos, Madrid, Spain). The 3 possible haplotypes will be associated with different fluorescent dyes to determine the identification of the genotypes: Val/Val, Val/Met, or Met/Met. The results are derived from a G→A substitution at the following sequence:

CCAGCGGATGGTGGATTTCGCTGGC [A/G] TGAAGGACAAGGTGTGCATGCCTGA

**REFERENCES**

1. Papanicolaou GD, McCabe SJ, Firrell J. The prevalence and characteristics of nerve compression symptoms in the general population. J Hand Surg [Am] 2001; 26: 460-6

Bland JD, Rudolfer SM. Clinical surveillance of carpal tunnel syndrome in two areas of the United Kingdom, 1991-2001. J Neurol Neurosurg Psychiatry 2003; 74: 1674-9.

Foley M, Silverstein B, Pollisar N. The economic burden of carpal tunnel syndrome: long term earnings of CTS claimants in Washington State. Am J Ind Med 2007; 50: 155-172

Page MJ, O’Connor D, Pitt V, Massy-Westropp N Exercise and mobilisation interventions for carpal tunnel syndrome. Cochrane Database Syst Rev 2012; 6: CD009899

Verdugo RJ, Salinas RA, Castillo JL, Cea JG. Surgical versus non-surgical treatment for carpal tunnel syndrome. Cochrane Database Syst Rev 2008; 4: CD001552

1. Shi Q, MacDemid JC. Is surgical intervention more effective than nonsurgical treatment for carpal tunnel syndrome?: A systematic review. J Orthop Surg Res 2011; 6: 17
2. Huisstede BM, Hoogvliet P, Randsdorp MS, Glerum S, van Middelkoop M, Koes BW. Carpal tunnel syndrome - Part I: effectiveness of nonsurgical treatments: a systematic review. Arch Phys Med Rehabil 2010; 91:981-1004
3. Jarvik JG, Comstock BA, Kliot M et al. Surgery versus non-surgical therapy for carpal tunnel syndrome: a randomised parallel-group trial. Lancet 2009; 374: 1074-1081
4. Gerritsen AA, de Vet HC, Scholten RJ, Bertelsmann FW, de Krom MC, Bouter LM. Splinting vs surgery in the treatment of carpal tunnel syndrome: a randomized controlled trial. JAMA 2002; 288: 1245-51
5. Ucan H, Yagci I, Yilmaz L, Yagmurlu F, Keskin D, Bodur H: Comparison of splinting, splinting plus local steroid injection and open carpal túnel release outcomes in idiopathic carpal tunnel syndrome. Rheumatol Int 2006; 27: 45-51.
6. Elwakil TF, Elazzazi A, Shokeir H: Treatment of carpal tunnel syndrome by low-level laser versus open carpal tunnel release. Lasers Med Sci 2007; 22: 265-270.
7. Fernández-de-las-Peñas C, De-la-Llave-Rincón AI, Fernández-Carnero J, Cuadrado ML, Arendt-Nielsen L, Pareja JA. Bilateral widespread mechanical pain sensitivity in carpal tunnel syndrome: evidence of central processing in unilateral neuropathy. Brain 2009; 132: 1472-9

Zanette G, Cacciatori C, Tamburin S. Central sensitization in carpal tunnel syndrome with extraterritorial spread of sensory symptoms. Pain 2010; 148: 227-36

1. De-la-Llave-Rincón AI, Fernández-de-las-Peñas C, Laguarta-Val S, Alonso-Blanco C, Martínez-Perez A, Arendt-Nielsen L, Pareja JA. Increased pain sensitivity is not associated with electrodiagnostic findings in women with carpal tunnel syndrome. Clin J Pain 2011; 27: 747-54

Nijs J, Van Houdenhove B, Oostendorp RA. Recognition of central sensitization in patients with musculoskeletal pain: Application of pain neurophysiology in manual therapy practice. Man Ther 2010; 15: 135-141.

Bialosky JE, Bishop MD, Price DD, Robinson ME, George SZ. The mechanisms of manual therapy in the treatment of musculoskeletal pain: a comprehensive model. Man Ther 2009; 14: 531-8

1. American Association of Electro-diagnostic Medicine, American Academy of Neurology, American Academy of Physical Medicine and Rehabilitation. Practice parameter: electro-diagnostic studies in carpal tunnel syndrome. Neurology 2002; 58: 1589-92

Padua L, Padua R, Aprile I, Tonali P. Italian multi-centre study of carpal tunnel syndrome: Differences in the clinical and neurophysiological features between male and female patients. J Hand Surg 1999; 24: 579-82

1. Moraska A, Chandler C, Edmiston-Schaetzel A, Franklin G, Calenda EL, Enebo B Comparison of a targeted and general massage protocol on strength, function, and symptoms associated with carpal tunnel syndrome: a randomized pilot study. J Altern Complement Med 2008; 14: 259-67

Butler DS. Mobilisation of the Nervous System Melbourne, Australia: Churchill Livingstone; 1991

Butler DS. The Sensitive Nervous System. Adelaide, Australia: Noigroup Publications; 2000

Scholten RJ, Mink van der Molen A, Uitdehaag BM, Bouter LM, de Vet HC. Surgical treatment options for carpal tunnel syndrome. Cochrane Database Syst Rev 2007; 4: CD003905.

1. Jensen MP, Turner JA, Romano JM, Fisher L. Comparative reliability and validity of chronic pain intensity measures. Pain 1999; 83: 157-62

Farrar JT, Young JP Jr., LaMoreaux L, Werth JL, Poole RM. Clinical importance of changes in chronic pain intensity measured on an 11-point numerical pain rating scale. Pain 2001; 94: 149-158

1. Rosales RS, Benseny E, Díez de la Lastra-Bosch I. Evaluation of the Spanish version of the DASH and carpal tunnel syndrome health-related quality of life instruments: cross cultural adaptation process and reliability. J Hand Surg 2002; 27A:334-43
2. Levine DW, Simmons B, Koris M, Daltroy L, Hohl G, Fossel A, Katz J. A self-administered questionnaire for the assessment of severity of symptoms and functional status in carpal tunnel syndrome. J Bone Joint Surg Am 1993; 75: 1585-92
3. Carvalho-Leite J, Jerosch-Herold C, Song F. A systematic review of the psychometric properties of the Boston Carpal Tunnel Questionnaire. BMC Musculoskeletal Disorders 2006; 7: 78
4. Kim JK, Jeon SH. Minimal clinically important differences in the Carpal Tunnel Questionnaire after carpal tunnel release. J Hand Surg Eur Vol 2013; 38: 75-9

Wyrwich K, Nienaber N, Tierney W, Wolinsky F. Linking clinical relevance and statistical significance in evaluating intra-individual changes in health-related quality of life. Med Care 1999; 37: 469-478

Jaeschke R, Singer J, Guyatt G. Measurement of health status: ascertaining the minimal clinically important difference. Controlled Clin Trials 1989; 10: 407-415

# Ghasemi-Rad M, Nosair E, Vegh A, Mohammadi A, Akkad A, Lesha E, Mohammadi MH, Sayed D, Davarian A, Maleki-Miyandoab T, Hasan A. A handy review of**carpal tunnel syndrome**: From anatomy to**diagnosis**and treatment. World J Radiol 2014; 6: 284-300

1. De-la-Llave-Rincón AI, Puentedura EJ, Fernández-de-las-Peñas C. New advances in the mechanisms and etiology of carpal tunnel syndrome. Discov Med 2012; 13: 343-8
2. Fernández-de-las-Peñas C, Ambite-Quesada S, Ortega-Santiago R, Martínez-Perez A, Díaz HF, Martínez-Martín J, Pareja JA. Catechol-O-methyltransferase Val158Met polymorphism is associated with pain and disability, but not widespread pressure pain sensitivity, in women with carpal Tunnel syndrome. Pain Physician 2013; 16: E591-60
3. Rakvåg TT, Klepstad P, Baar C et al. The Val158Met polymorphism of the human catechol-O-methyltransferase (COMT) gene may influence morphine requirements in cancer pain patients. Pain 2005; 116:73-8
